# High-intensity interval training using electrical stimulation ameliorates muscle fatigue in chronic kidney disease-related cachexia by restoring mitochondrial respiratory dysfunction

**DOI:** 10.3389/fphys.2024.1423504

**Published:** 2024-06-26

**Authors:** Hiroyori Fusagawa, Tatsuya Sato, Takashi Yamada, Azuma Naito, Nao Tokuda, Nao Yamauchi, Nobutoshi Ichise, Toshifumi Ogawa, Takuro Karaushi, Atsushi Teramoto, Noritsugu Tohse

**Affiliations:** ^1^ Department of Cellular Physiology and Signal Transduction, Sapporo Medical University School of Medicine, Sapporo, Japan; ^2^ Department of Orthopaedic Surgery, Sapporo Medical University School of Medicine, Sapporo, Japan; ^3^ Department of Cardiovascular, Renal and Metabolic Medicine, Sapporo Medical University School of Medicine, Sapporo, Japan; ^4^ Graduate School of Health Sciences, Sapporo Medical University, Sapporo, Japan

**Keywords:** chronic kidney disease, cachexia, muscle endurance, high-intensity interval training, mitochondria

## Abstract

**Background:**

Exercise, especially high-intensity interval training (HIIT), can increase mitochondrial respiratory capacity and enhance muscular endurance, but its systemic burden makes it difficult to safely and continuously prescribe for patients with chronic kidney disease (CKD)-related cachexia who are in poor general condition. In this study, we examined whether HIIT using electrical stimulation (ES), which does not require whole-body exercise, improves muscle endurance in the skeletal muscle of 5/6 nephrectomized rats, a widely used animal model for CKD-related cachexia.

**Methods:**

Male Wistar rats (10 weeks old) were randomly assigned to a group of sham-operated (Sham) rats and a group of 5/6 nephrectomy (Nx) rats. HIIT was performed on plantar flexor muscles *in vivo* with supramaximal ES every other day for 4 weeks to assess muscle endurance, myosin heavy-chain isoforms, and mitochondrial respiratory function in Nx rats. A single session was also performed to identify upstream signaling pathways altered by HIIT using ES.

**Results:**

In the non-trained plantar flexor muscles from Nx rats, the muscle endurance was significantly lower than that in plantar flexor muscles from Sham rats. The proportion of myosin heavy chain IIa/x, mitochondrial content, mitochondrial respiratory capacity, and formation of mitochondrial respiratory supercomplexes in the plantaris muscle were also significantly decreased in the non-trained plantar flexor muscles from Nx rats than compared to those in plantar flexor muscles from Sham rats. Treatment with HIIT using ES for Nx rats significantly improved these molecular and functional changes to the same degrees as those in Sham rats. Furthermore, a single session of HIIT with ES significantly increased the phosphorylation levels of AMP-activated protein kinase (AMPK) and p38 mitogen-activated protein kinase (MAPK), pathways that are essential for mitochondrial activation signaling by exercise, in the plantar muscles of both Nx and Sham rats.

**Conclusion:**

The findings suggest that HIIT using ES ameliorates muscle fatigue in Nx rats via restoration of mitochondrial respiratory dysfunction with activation of AMPK and p38 MAPK signaling. Our ES-based HIIT protocol can be performed without placing a burden on the whole body and be a promising intervention that is implemented even in conditions of reduced general performance status such as CKD-related cachexia.

## Introduction

Chronic kidney disease (CKD) is an increasing health problem, and the prevalence of CKD is estimated to be more than 10% of the general population worldwide, amounting to over 800 million individuals (Kovesdy, 2022). CKD is a complex disease that begins with decreased kidney function and induces multiple organ dysfunction. Among the various complications of CKD, cachexia has been reported to be associated with a poor prognosis and there is still no effective treatment strategy (Koppe et al., 2019). CKD-related cachexia is often accompanied by sarcopenia, and a strong association between the degree of renal failure and sarcopenia has been suggested (Chatzipetrou et al., 2022). It has been reported that renal failure causes muscle disturbances with mitochondrial dysfunction caused by intramitochondrial accumulation of uremic toxins, production of reactive oxygen species, and inflammation-induced mitochondrial DNA damage (Ertuglu et al., 2022), resulting in a decrease in muscle endurance (Tamaki et al., 2014). We also found that muscle endurance of fast-twitch muscles in 5/6 nephrectomized rats is reduced in the early stages of the disease, accompanied by a decrease in muscle mitochondrial function. (Fusagawa et al., 2023). Muscle endurance has been reported to correlate more with lower extremity function than maximal exerted muscle strength (Roshanravan et al., 2017), and it is clear that muscle fatigability is what should be addressed in CKD to prevent the worst-case scenario of falls, fractures, and bedridden status from occurring. In addition, while disuse muscle atrophy caused by casting, unloading, and other conditions causes muscle atrophy mainly in slow-twitch muscles, cachexia causes damage mainly in fast-twitch muscles, and it is known that fast-twitch muscle fibers are also markedly atrophied in CKD (Sabatino et al., 2021). Decreased endurance of fast-twitch muscles causes muscle weakness in the early phase of endurance exercise, which impairs balance during walking and leads to falls (Parijat and Lockhart, 2008; Inoue et al., 2013). Our previous *ex vivo* experiments with fast-twitch muscle have shown that muscle force decline in the early phase of endurance exercise in CKD is influenced by fast-twitch muscle fibers (Fusagawa et al., 2023). Accordingly, in CKD-related cachexia, it is necessary to focus on improvement in muscle endurance that occurs in fast-twitch muscles with therapeutic intervention methods against mitochondrial dysfunction.

Exercise therapy in patients with CKD was believed to increase proteinuria and worsen kidney damage, but it has recently become widely recommended due to the lack of clear evidence that it worsens renal function. However, conventional endurance training to improve muscle endurance is difficult for patients with severe renal failure to continuously perform. High-intensity interval training (HIIT) consists of intense exercise interspersed by short periods of recovery and it has been shown to be more effective way than moderate-intensity continuous training for improving mitochondrial function and fatigue resistance in muscles of both healthy individuals and individuals with diseases (Torma et al., 2019). However, HIIT is a cruel order to prescribe for renal failure patients as it is more demanding on the entire body than a usual exercise protocol. To address this issue, we have proposed a HIIT method using electrical stimulation (ES) that provides exercise load to a local area and is expected to be effective with less concern about systemic adverse events. In fact, we have obtained results showing improvement of muscle endurance in normal, arthritic, and muscular dystrophic mice (Yamada et al., 2021; Yamada et al., 2022; Yamauchi et al., 2023). Based on such results, we hypothesized that HIIT with ES is safe and effective for skeletal muscle in CKD. Therefore, the purpose of this study was to investigate the effects of HIIT with ES on muscle endurance and cellular adaptation in a 5/6 nephrectomized rat model.

## Methods

### Experimental approval and 5/6 nephrectomized rats

All experimental protocols were reviewed and approved by the Ethics Committee on Animal Experiments of Sapporo Medical University (No. 20–076, Sapporo, Japan). Animal care was performed in strict accordance with institutional guidelines. Male Wistar rats (10 weeks old, *n* = 40) were supplied by Sanyo Labo Service (Sapporo, Japan). The rats were housed in an environmentally controlled room (24°C ± 2°C, 12 h: 12 h light-dark cycle) and given food and water *ad libitum*. After acclimation, they were allocated to a group of sham-operated control (Sham) rats or a group of 5/6 rephrectomy (Nx) rats for inducing CKD. The 5/6 nephrectomy was performed on the rats by a two-step procedure that reduces the original renal mass by five-sixths as previously described (Nishizawa et al., 2016). Briefly, in the first step as 1/2 nephrectomy, the right kidney was removed via a right flank incision under anesthesia with 2% inhaled isoflurane. At 1 week after the procedure, 2/3 of the remaining kidney was removed by resecting the upper and lower poles of the left kidney via a left flank incision. Bleeding was controlled with an adhesive agent for tissue (Spongel, LTL Pharma, Japan). Sham rats only received capsulotomy with the same operation duration as that in Nx rats. 8 weeks after the second operation, we confirmed a decline of muscle endurance in plantar flexor muscles from Nx rats (Fusagawa et al., 2023).

### Experimental design

To assess the effect of HIIT using ES on the skeletal muscle of Nx rats, we performed two separate experiments. In the first experiment, Nx rats were subjected to 4 weeks of HIIT, and the muscle physiological and molecular biological adaptations were evaluated. In the second experiment, sampling immediately after one HIIT session was performed to examine the cellular signaling underlying the adaptations. All HIIT training and muscle endurance assessments utilized Isometric contraction with the rat’s ankle joint in a fixed position.

### Experiment 1

**FIGURE 1:**
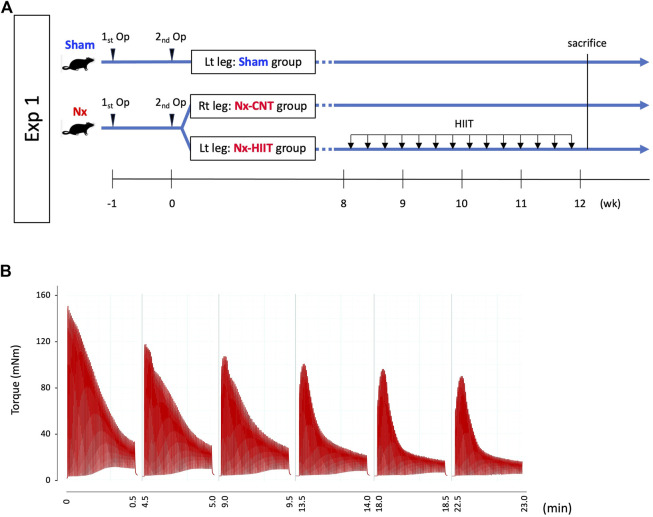
Experimental overview and high-intensity interval training (HIIT) with electrical stimulation (ES). **(A)** In experiment (Exp) 1, muscle endurance of plantar flexor muscles and intracellular events were evaluated in sham-operated (Sham) rats and 5/6 nephrectomy (Nx) rats with and without subsequent HIIT. HIIT started 8 weeks after the second nephrectomy operation and was performed with ES on the left leg (Nx-HIIT) every other day for a total 14 sessions. The right leg (NX-CNT) served as a non-training Nx control. **(B)** Typical torque traces of a HIIT session.

To examine the effect of HIIT on muscle endurance in Nx rats, male Wistar rats were randomly assigned to a group of Sham rats (*n* = 14) and a group of Nx-HIIT rats (*n* = 14). In each of the Nx-HIIT rats, HIIT was performed on the left leg (referred to as the Nx-HIIT group), and the right leg served as a non-training Nx control (Nx-CNT group). Sham rats had no intervention during the HIIT period and the left leg was used as the Sham group. HIIT was started 8 weeks after the second nephrectomy operation and was carried out every other day for a total of 14 sessions over a period of 4 weeks (Figure 1A). Under isoflurane anesthesia, mice were placed supine on a platform with the foot secured to a footplate connected to a torque sensor (S-14154, Takei Scientific Instruments) at an angle of 0° dorsiflexion (i.e., 90° relative to the tibia). The plantar flexor muscles were activated by supramaximal (45 V, 0.5 ms) monophasic rectangular current pulses via a pair of surface electrodes. The stimulation scheme was designed to mimic the activation pattern during all-out cycling bouts, i.e., 0.25-s contractions produced every 0.5 s (Place et al., 2015; Yamada et al., 2021). Typical torque traces during a HIIT session are shown in Figure 1B. Each session consisted of six sets of 60 contractions at 4-m intervals. Twenty-four hours after the last HIIT session, *in vivo* muscle endurance of the plantar flexor muscles in each group was measured by repeated electrical stimulation. Twenty-four hours after the measurement of muscle endurance (i.e., 48 h after the last HIIT session), rats were sacrificed by cervical dislocation after blood sampling under isoflurane anesthesia and the plantaris muscles were frozen in liquid nitrogen for biochemical analyses. To evaluate the effect of HIIT on renal function, four additional Nx rats as Nx-CNT rats were added to the study group and blood samples were taken after 4 weeks of no HIIT in both legs starting 8 weeks after the second surgery. For blood sampling, cardiac puncture with a 20-gauge needle was performed to collect blood. Estimated glomerular filtration rate (eGFR) (µL/min) from blood sample data was calculated using the following equations (Besseling et al., 2021): plasma creatinine < 52 µmol/L: eGFR = 880 × *W* ^0.695^ × *C* ^−0.660^ × *U* ^−0.391^, plasma creatinine ≥ 52 µmol/L: eGFR = 5,862 × *W* ^0.695^ × *C* ^−1.150^ × *U* ^−0.391^. *W* is weight (g), *C* is creatinine concentration (mmol/L), and *U* is urea (mmol/L).

### Experiment 2

To investigate cellular signal underlying the HIIT-induced physiological adaptations, male Wistar rats (*n* = 12) were randomly divided into a group of Sham rats and a group of Nx rats (*n* = 6 in each group). In both Sham and Nx rats, HIIT was performed on the left leg (referred to as the Sham-HIIT and Nx-HIIT groups), and the right leg served as a non-HIIT control (Sham-CNT and Nx-CNT groups). Immediately after one HIIT session of left leg, rats were killed by rapid cervical dislocation under isoflurane anesthesia, and the muscles were subsequently isolated. The phosphorylation levels of AMPKα Thr172, CaMKII Thr286, and p38 MAPK Thr180/Tyr182 were investigated in the plantaris muscles of each group.

### Measurement of *in vivo* muscle endurance

In experiment 1, 24 h after the last HIIT session, *in vivo* fatigue resistance of the plantar flexor muscles in each group was measured by 100 repeated 350 ms, 70 Hz tetani given at an interval of 2 s using the same apparatus settings as those for HIIT with ES.

### Biochemical analyses

To obtain whole muscle protein lysates, pieces of the plantaris muscle were homogenized in ice-cold homogenizing buffer (30 µL/mg wet wt) consisting of (in mM): Tris maleate, 10; NaF, 35; NaVO_4_, 1; 1% Triton × 100 (vol/vol), and 1 tablet of protease inhibitor cocktail (Roche) per 50 mL. Muscle protein lysates were stored at −80°C until the following biochemical experiments.

### Separation of myosin heavy chain isoforms

Aliquots of the homogenized plantaris muscles were used for myosin heavy chain (MHC) electrophoresis as previously described in detail (Wada et al., 1996; Yamauchi et al., 2023). In brief, after the sample is lysed as in the Biochemical analysis section, Aliquots of the whole muscle homogenates (5 μg) were diluted with SDS-sample buffer (mM): Tris–HCl, 62.5; 2% SDS (w/v); 10% glycerol (v/v); 5% 2-mercaptoethanol (v/v); 0.02% bromophenol blue (w/v). Proteins were applied to a 6% polyacrylamide slab gel. Electrophoresis was run at 4°C for 24 h at 160 V and the gel was stained with Coomassie brilliant blue. Images of gels were densitometrically quantified with ImageJ.

### Mitochondrial enzyme activity assay

The maximal activities of citrate synthase (CS) were determined in whole muscle homogenates. In brief, the whole plantaris muscles were homogenized in ice-cold 100 mM potassium phosphate buffer (100 μL/mg wet wt), and maximal CS activity was measured spectrophotometrically as described previously (Smith, 1955).

### Measurement of mitochondrial respiratory capacity

Oxygen consumption rates of freshly isolated mitochondria from the plantaris muscle were measured using Seahorse XFe96 Bioanalyzer as previously described with slight modifications (Sato et al., 2018). In brief, muscles that had been quickly excised from rats were cut into small pieces in ice-cold fiber relaxation buffer (100 mM KCl; 5 mM EGTA; 5 mM HEPES, pH adjusted to 7.0 with KOH). The muscle pieces were then homogenized in HES buffer (5 mM HEPES; 1 mM EDTA; 250 mM sucrose, pH adjusted to 7.4 with KOH) using a Dounce homogenizer. The homogenate was centrifuged for 10 m at 500 *g* twice and the supernatant was centrifuged at 9,000 *g* for 15 m at 4°C. The pellet including crude mitochondria was re-suspended in MAS buffer (70 mM sucrose; 220 mM mannitol; 10 mM KH_2_PO_4_; 5 mM MgCl_2_; 2 mM HEPES; 1 mM EGTA; 0.2% fatty acid-free BSA, pH adjusted to 7.4 with KOH). The protein concentration was measured using a BCA Protein Quantification Kit (Takara-bio, Japan). Since the buffer contains BSA, the mitochondrial protein concentration was determined by subtracting the background BSA protein content. Equal amounts of mitochondria in MAS buffer (10 µg) were loaded in each well for pyruvate/malate-driven respiration and succinate-driven respiration. The plates were centrifuged at 1,400 *g* for 20 m. Following centrifugation, MAS buffer containing 5 mM pyruvate and 5 mM malate or 5 mM sodium succinate and 2 µM rotenone was added to a final volume of 180 µL and incubated for 8 m at 37°C without CO_2_. Oxygen tension was measured in the Seahorse XFe96 Bioanalyzer at baseline and following injections of 20 mM ADP, 10 μM oligomycin, and 5 μM rotenone/antimycin.

### Western blot

The homogenized aliquot was centrifuged at 14,000 *g* for 15 m at 4°C to obtain the supernatant. The protein content was determined using a bicinchoninic acid (BCA) Protein Quantification Kit (Takara-bio, Japan). Equal amounts of protein were resolved and loaded using NuPAGE Novex 4%–12% Bis-Tris midi gels (Thermo Fisher Scientific, Waltham, MA, United States) and transferred to nitrocellulose membranes. Total proteins on the membrane were visualized by 0.1% (wt/vol) of Ponceau S in 5% acetic acid. After blocking with Tris-buffered saline (TBS) containing 0.005% Tween 20% and 5% milk, the membranes were incubated overnight with primary antibodies against oxidative phosphorylation (OXPHOS) antibody cocktail (ab110413, Abcam, Cambridge, United Kingdom), NDUFS1 (sc271387, Santa Cruz biotechnology, Santa Cruz, CA, United States), cytochrome c oxidase (COX) IV (ab16056, Abcam), prohibitin (ab28172, Abcam), anti-phospho-AMPKα Thr172 (#2531, Cell Signaling, Beverly, MA, United States), anti- AMPKα (#2532, Cell Signaling), anti-phospho-CaMKII Thr286 (#12716, Cell Signaling), anti-CaMKII (611,292, BD Biosciences, San Jose, CA, United States), anti-phospho-p38 MAPK (#4511, Cell Signaling), and anti-p38 MAPK (#9212, Cell Signaling). Horseradish peroxidase-conjugated anti-mouse or anti-rabbit secondary antibodies were purchased from Bio-Rad (Hercules, CA, United States). The blots were developed using Pierce ECL reagent (Thermo Fisher Scientific, Waltham, MA, United States). Images were photographed and processed using a ChemiDoc XRS þ System with Image Lab software (Bio-Rad, Hercules, CA, United States). Intensities of individual bands were normalized by total proteins detected by Ponceau S staining and quantified by using ImageJ software.

### Blue native gel electrophoresis on isolated mitochondria

Blue native gel electrophoresis was performed as previously described with slight modifications (Sato et al., 2018). In brief, plantaris muscles that had been quickly excised from rats were cut into small pieces on ice and mitochondria were isolated from the muscle using Mitochondria Isolation Kit (Pierce, Rockford, IL, United States). Isolated mitochondria were then extracted with 2.0% digitonin. The protein concentration was determined using BCA Protein Quantification Kit (Pierce, Rockford). Equal mitochondrial proteins (5 μg) were loaded onto a Native PAGE Bis-Tris Protein Gel (Life Technologies, CA, United States) according to the manufacturer’s instructions. Gels were stained by Coomassie Brilliant Blue R-250 (Cosmo Bio, Tokyo, Japan) for quantification of the amount of mitochondrial respiratory supercomplexes (SCs).

### Statistics

Data are presented as means ± SD and as individual values. The normality of the data was tested using the Shapiro-Wilk normality test. Results were analyzed by Welch’s *t*-test for experiments comparing two groups. When more than two groups were compared, one-way ANOVA followed by Tukey’s test or two-way ANOVA followed by Sidak’s test was used to analyze differences between groups. All statistics were performed on GraphPad Prism eight and differences were considered statistically significant at *p* < 0.05 (*).

## Results

### HIIT with ES improves muscle endurance in plantar flexor muscles of CKD rats

**FIGURE 2:**
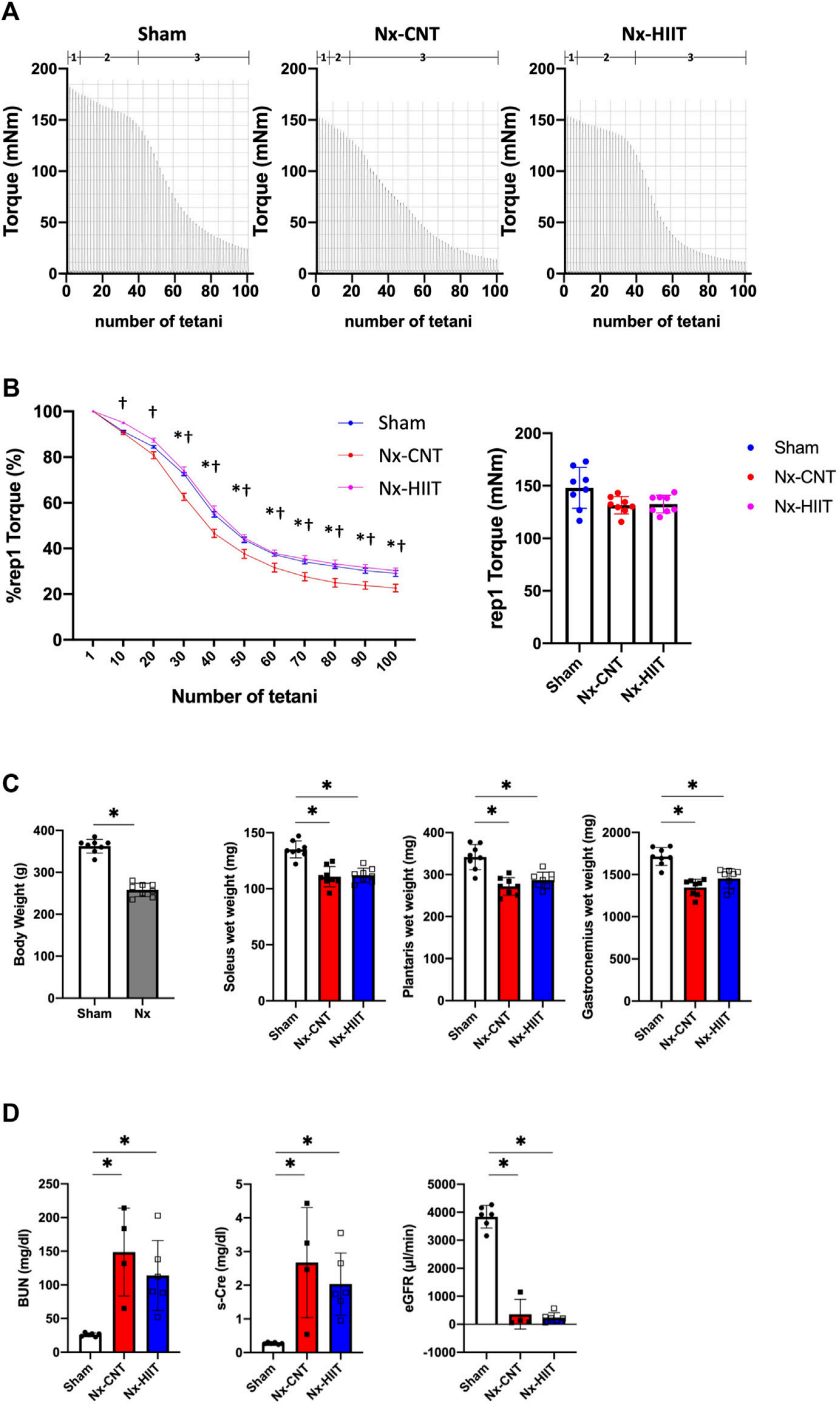
High-intensity interval training (HIIT) with electrical stimulation improves muscle endurance in skeletal muscle of 5/6 nephrectomy (Nx) rats. **(A)** Representative torque records during *in vivo* fatigue stimulation (70 Hz, 350 m tetani every 2 s) of plantar flexor muscles from sham-operated (Sham) rats (*n* = 8) and Nx rats with HIIT (*n* = 8) or without HIIT (*n* = 8). Numbers above the *x*-axis refer to fatigue phases as explained in the text: an initial force decrease of ∼10% over ten tetani (phase 1), followed by a subacute period of slow force decline (phase 2), and finally a more rapid force decrease (phase 3). **(B)** The left panel showed the mean (± SEM) relative tetanic torque during fatiguing stimulation. Torque in the first tetanus was set to 100% in each muscle. Two-way repeated-measures ANOVA with Sidak’s *post hoc* test was performed. *p < 0.05 Sham vs. Nx-CNT, ^#^ *p* < 0.05 Sham vs. Nx-HIIT, ^†^ *p* < 0.05 Nx-CNT vs. Nx-HIIT. The right panel showed the mean (± SD) absolute torque of the first tetanus. One-way ANOVA followed by Tukey’s *post hoc* test showed no significant differences between groups. **(C)** Body weights of Sham rats (*n* = 8) and Nx rats (*n* = 8). *p < 0.05 with Welch’s *t*-test for comparison of two groups. The muscle wet weights of plantar flexor muscles including soleus, plantaris, and gastrocnemius muscles from Sham rats (*n* = 8) and Nx rats with HIIT (*n* = 8) or without HIIT (*n* = 8). *p < 0.05 with one-way ANOVA followed by Tukey’s *post hoc* test for comparison of groups. **(D)** Serum parameters in Sham, Nx-CNT and Nx-HIIT rats. *p < 0.05 vs. Sham with one-way ANOVA followed by Tukey’s test for comparison of three groups, Sham vs. Nx-CNT vs. Nx-HIIT rats. BUN, blood urea nitrogen; s-Cre, serum creatinine; eGFR, estimated glomerular filtration rate. Data are shown as means ± SD.

At 8 weeks after 5/6 nephrectomy, we observed a decrease in muscle endurance in both legs of Nx rats as previously reported (Fusagawa et al., 2023). Consequently, we initiated the HIIT protocol in the left leg of Nx rats, designated as the Nx-HIIT group. Typical torque traces during a HIIT session are shown in Figure 1B. In experiment 1, after the HIIT for 4 weeks, measurement of *in vivo* muscle endurance showed that the duration of phase 2 in the Nx-CNT group had clearly been shortened or had almost disappeared compared to that in the Sham and Nx-HIIT groups: an initial force decrease of ∼10% over ten tetani (phase 1), followed by a subacute period of slow force decline (phase 2), and finally a more rapid force decrease (phase 3) (Allen et al., 2008) (Figure 2A). Statistically, the Nx-CNT group showed less muscle endurance than that in the Sham group, while the Nx-HIIT group showed significantly higher muscle endurance than that in the Nx-CNT group (Figure 2B). In this process, we found no significant difference in the one-way ANOVA comparison of the starting absolute torque among the three groups (Sham versus Nx-CNT, *p* = 0.0512; Sham versus Nx-HIIT, *p* = 0.0717; Nx-CNT versus Nx-HIIT, *p* = 0.9848), confirming that we can accurately compare the muscle endurance of these three groups. There was a significant difference in body weight between Sham rats and Nx-HIIT rats (362.5 ± 16.5 g versus 258.1 ± 15.8 g, *p* = 0.0002). The soleus, plantaris, and gastrocnemius muscle weights were 18%, 20%, and 21% lower, respectively, in the Nx-CNT group than in the Sham group (110.9 ± 9.0 versus 135.2 ± 7.6 mg, *p* < 0.0001; 271.9 ± 21.0 versus 342.0 ± 30.0 mg, *p* < 0.0001; 1,348 ± 97.3 versus 1,714 ± 106.0 mg, *p* < 0.0001), and this was not ameliorated by HIIT (112.0 ± 6.4 mg, *p* = 0.9529; 287.1 ± 18.7 mg, *p* = 0.4211; 1,454 ± 114.5 mg, *p* = 0.1390) (Figure 2C). Serum tests showed that Nx-CNT rats and Nx-HIIT rats had significantly higher levels of blood urea nitrogen (5.7-fold and 4.4-fold higher, respectively) and serum creatinine (9.5-fold and 7.25-fold higher, respectively) and significantly lower levels of eGFR (0.09-fold and 0.06-fold lower, respectively) than those in Sham rats (Figure 2D). There were no significant differences in serum parameters of renal function between Nx-CNT and Nx-HIIT rats. These findings indicate that the HIIT with ES protocol used in the present study has the potential to safely improve muscle endurance without worsening renal function.

### HIIT with ES prevents the change of fiber type within fast-twitch muscles of CKD rats

**FIGURE 3:**
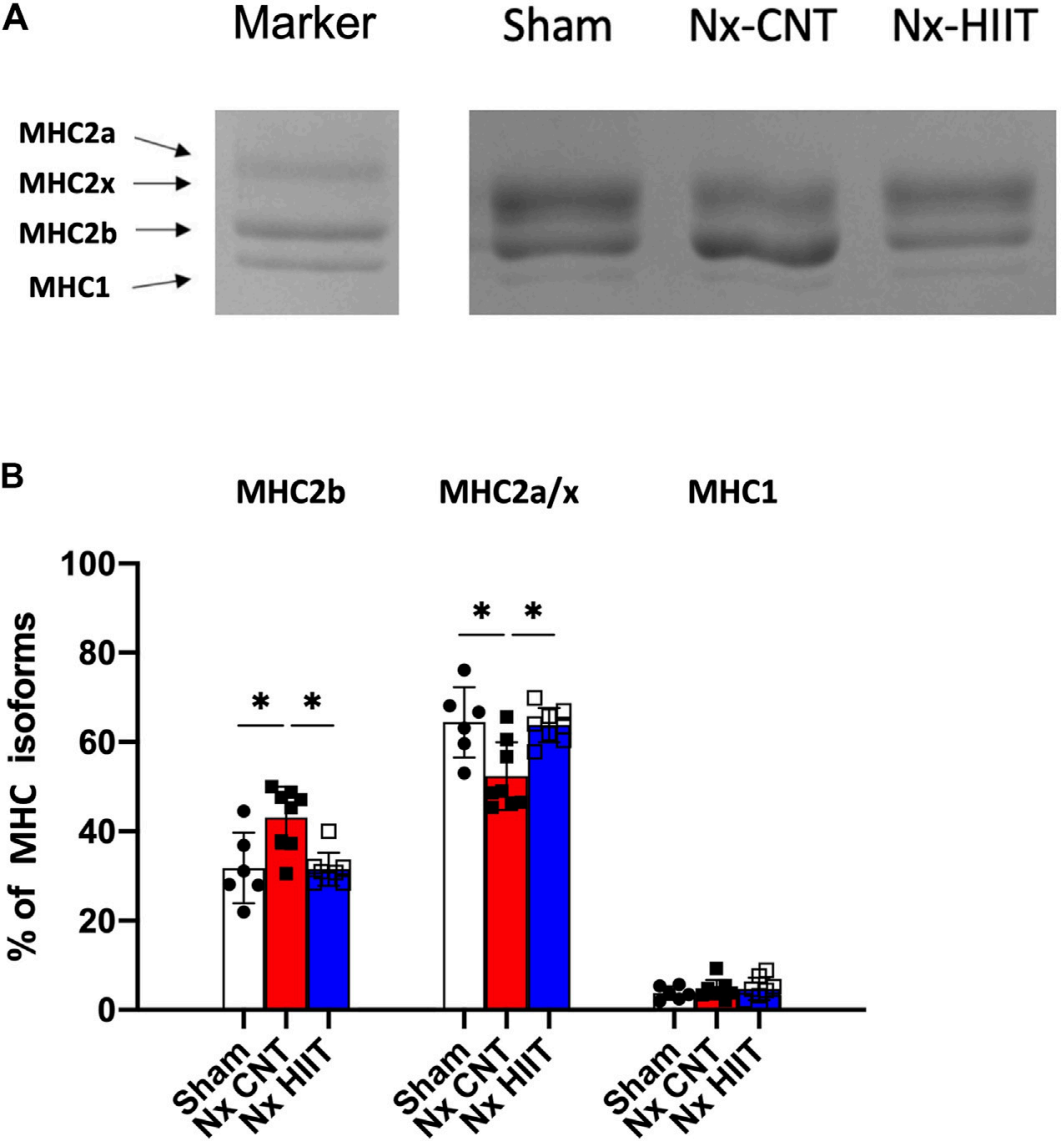
High-intensity interval training (HIIT) with electrical stimulation suppresses the increase in the percentage of glycolytic myosin heavy chain (MHC) within fast-twitch muscle of 5/6 nephrectomy (Nx) rats. **(A)** Blots show representative MHC isoforms separated by electrophoresis. **(B)** Mean ± SD percentage distribution of MHC isoforms in plantaris muscles from sham-operated (Sham) rats (*n* = 8) and Nx rats with HIIT (*n* = 8) or without HIIT (*n* = 8). In the marker lane, a mixture of a fast-twitch extensor digitorum longus muscle and a slow-twitch soleus muscle isolated from rats was applied as an indicator of each isoform. *p < 0.05 with one-way ANOVA followed by Tukey’s *post hoc* test for comparison of groups. Data are shown as means ± SD.

Previous studies showed that rodents in which CKD was induced exhibit a slow to fast fiber-type transformation in fast-twitch muscle (Tamaki et al., 2014; Acevedo et al., 2015), which may be associated with reduced muscle endurance. Electrophoresis of MHCs in the present study revealed that the proportion of glycolytic fibers (MHC2b) was significantly increased in the Nx-CNT group compared to that in the Sham group (43.1% ± 7.0% versus 31.8% ± 7.9%, *p* = 0.0094) and HIIT suppressed this change induced by Nx (31.5% ± 3.7%, *p* = 0.0043) (Figures 3A, B). Therefore, the changes from MHC2a/x to MHC2b within the fast-twitch muscle may contribute to the observed early decline in muscle endurance.

### HIIT with ES ameliorates the decrease of mitochondrial content and respiratory capacity in muscle of CKD rats

**FIGURE 4:**
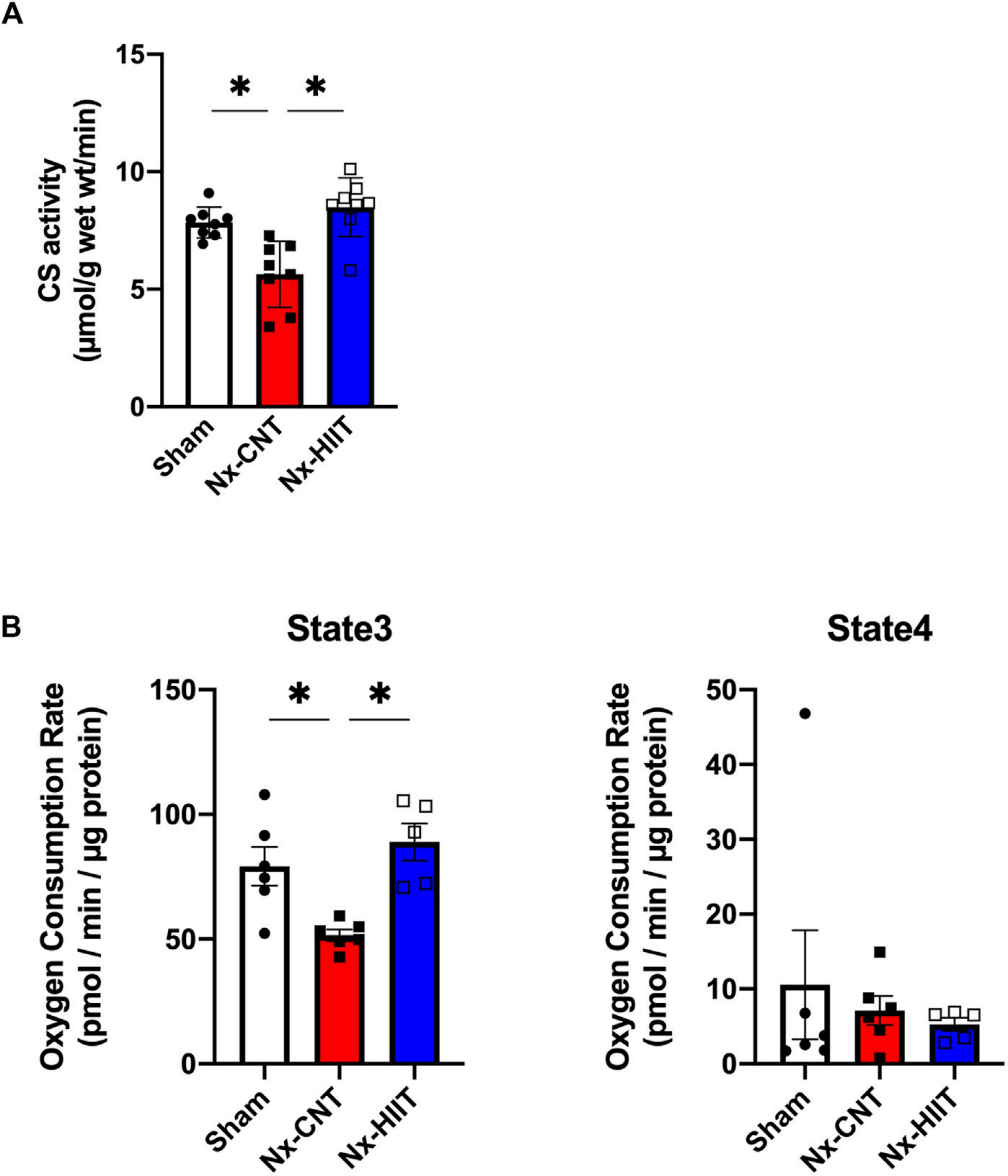
High-intensity interval training (HIIT) with electrical stimulation restores mitochondrial content and function in skeletal muscle of 5/6 nephrectomy (Nx) rats. **(A)** Citrate synthase (CS) activity in plantaris muscles from sham-operated (Sham) rats (*n* = 8) and Nx rats with HIIT (*n* = 8) or without HIIT (*n* = 8). **(B)** Pyruvate/malate-driven oxygen consumption rates determined by the Seahorse XFe96 analyzer in mitochondria isolated from plantaris muscles from sham-operated (Sham) rats (*n* = 6) and Nx rats with HIIT (*n* = 5) or without HIIT (*n* = 6). State three respiration indicates oxygen consumption with ADP and state four respiration indicates respiration with oligomycin. *p < 0.05 with one-way ANOVA followed by Tukey’s *post hoc* test for comparison of groups. Data are shown as means ± SD.

Mitochondria play a central role in energy metabolism and are key determinants of endurance performance. CS activity, which is widely used as a biomarker of mitochondrial content, was significantly lower in the plantaris muscle in the Nx-CNT group than in the plantaris muscle in the Sham group (5.6 ± 1.4 versus 7.8 ± 0.6 µmol/g wet weight/min, *p* = 0.0028) (Figure 4A). Importantly, the Nx-HIIT group showed restoration of the enzyme activity (8.5 ± 1.3 µmol/g wet weight/min, *p* = 0.0002), indicating that HIIT increased the mitochondrial content in the plantaris muscle in the Nx-HIIT group. Additionally, mitochondrial respiratory function normalized by mitochondrial protein content was analyzed using the Seahorse XFe96 Analyzer in the freshly isolated mitochondria. Pyruvate/malate-driven state three respiration, which represents oxygen consumption capacity in the presence of ADP, was significantly decreased in the Nx-CNT group compared to that in the Sham group (51.5 ± 5.7 versus 79.2 ± 19.1 pmol/min/µg protein, *p* = 0.0157), whereas state four (oligomycin-induced leak) respiration was unchanged (7.1 ± 4.7 versus 10.6 ± 17.9 pmol/min/µg protein, *p* = 0.8531) (Figure 4B). Notably, state three respiration was significantly recovered in the Nx-HIIT group, although state four respiration was unchanged (88.9 ± 16.7 pmol/min/µg protein, *p* = 0.0026; 5.3 ± 1.9 pmol/min/µg protein, *p* = 0.9585).

### HIIT with ES increases the amount of mitochondrial respiratory complexes and supercomplex formation in muscle of CKD rats

**FIGURE 5:**
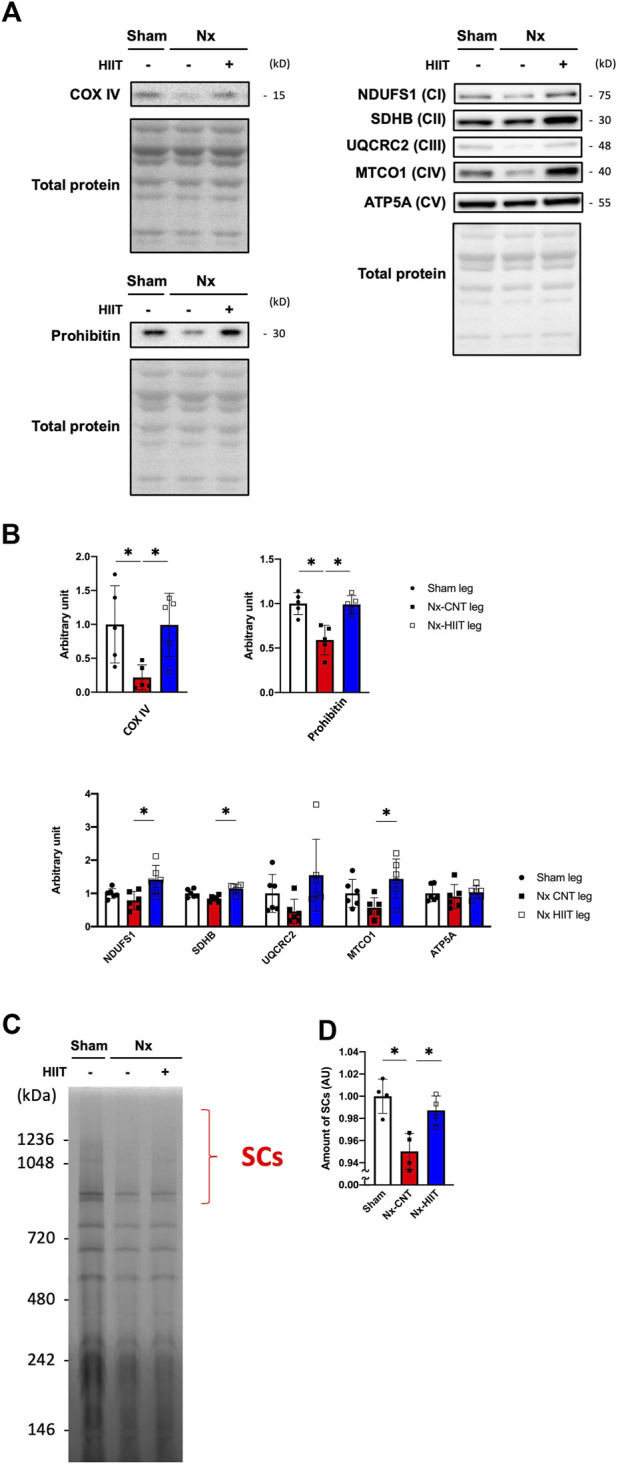
High-intensity interval training (HIIT) with electrical stimulation increases the content of mitochondrial respiratory chain complexes and supercomplex formation in skeletal muscle of 5/6 nephrectomy (Nx) rats. **(A)** Representative Western blots illustrating the levels of COX IV, prohibitin, complex I (CI) subunit (NDUFS1), CII subunit (SDHB), CIII subunit (UQCRC2), CIV subunit (MTCO1), and CV subunit (ATP5) in plantaris muscles. **(B)** The levels of COX IV, prohibitin, CI, CII, CIII, CIV, and CV in plantaris muscles from sham-operated (Sham) rats (*n* = 5–6) and Nx rats with HIIT (*n* = 5–6) or without HIIT (*n* = 5–6) were normalized by total proteins seen in the stain-free gels. **(C)** Representative BN-PAGE images and amount of supercomplexes (SCs). **(D)** The levels of SCs in plantaris muscles from Sham rats (*n* = 4) and Nx rats (*n* = 4) were normalized by the total proteins seen in the same lane. *p < 0.05 with one-way ANOVA followed by Tukey’s post hoc test for comparison of groups. Data are shown as means ± SD.

In agreement with the CS activity being decreased in the Nx-CNT group and increased in the Nx-HIIT group, COX IV and prohibitin, which are known as mitochondrial markers, showed significant decreases in the Nx-CNT group compared to those in the Sham group (COX IV: *p* = 0.0380; prohibitin: *p* = 0.0010) and significant increases in the Nx-HIIT group compared to those in the Nx-CNT group (COX IV: *p* = 0.0398; prohibitin: *p* = 0.0012) (Figures 5A, B). Furthermore, since the results obtained by using the Seahorse analyzer showed improved respiratory capacity per mitochondrial protein by HIIT, the amount of mitochondrial respiratory chain (MRC) complexes and supercomplex formation, which has been shown to enhance respiratory chain activity through spatial restriction of electron carrier diffusion (Acín-Pérez et al., 2008), were investigated. Although no MRC complexes showed a significant decrease in the Nx-CNT group compared to those in the Sham group (NDUFS1: *p* = 0.4668; SDHB: *p* = 0.0797; UQCRC2: *p* = 0.4266; MTCO1: *p* = 0.2629; ATP5A: *p* = 0.8285), NDUFS1, SDHB, and MTCO1 were significantly increased in the Nx-HIIT group compared to those in the Nx-CNT group (NDUFS1: *p* = 0.0072; SDHB: *p* = 0.0010; UQCRC2: *p* = 0.0529; MTCO1: *p* = 0.0130; ATP5A: *p* = 0.7036) (Figures 5A, B). The overall content of SCs in isolated mitochondria was decreased in the Nx-CNT group compared to that in the Sham group (*p* = 0.0029), but HIIT attenuated the decreased formation of respiratory SCs (*p* = 0.0169) (Figures 5C, D). These findings suggested that HIIT with ES enhances muscle endurance in CKD rats by restoring not only mitochondrial content but also oxidative phosphorylation capacity with improved mitochondrial respiratory supercomplex formation.

### Phosphorylation levels of signaling proteins are increased after a single bout of HIIT with ES

**FIGURE 6:**
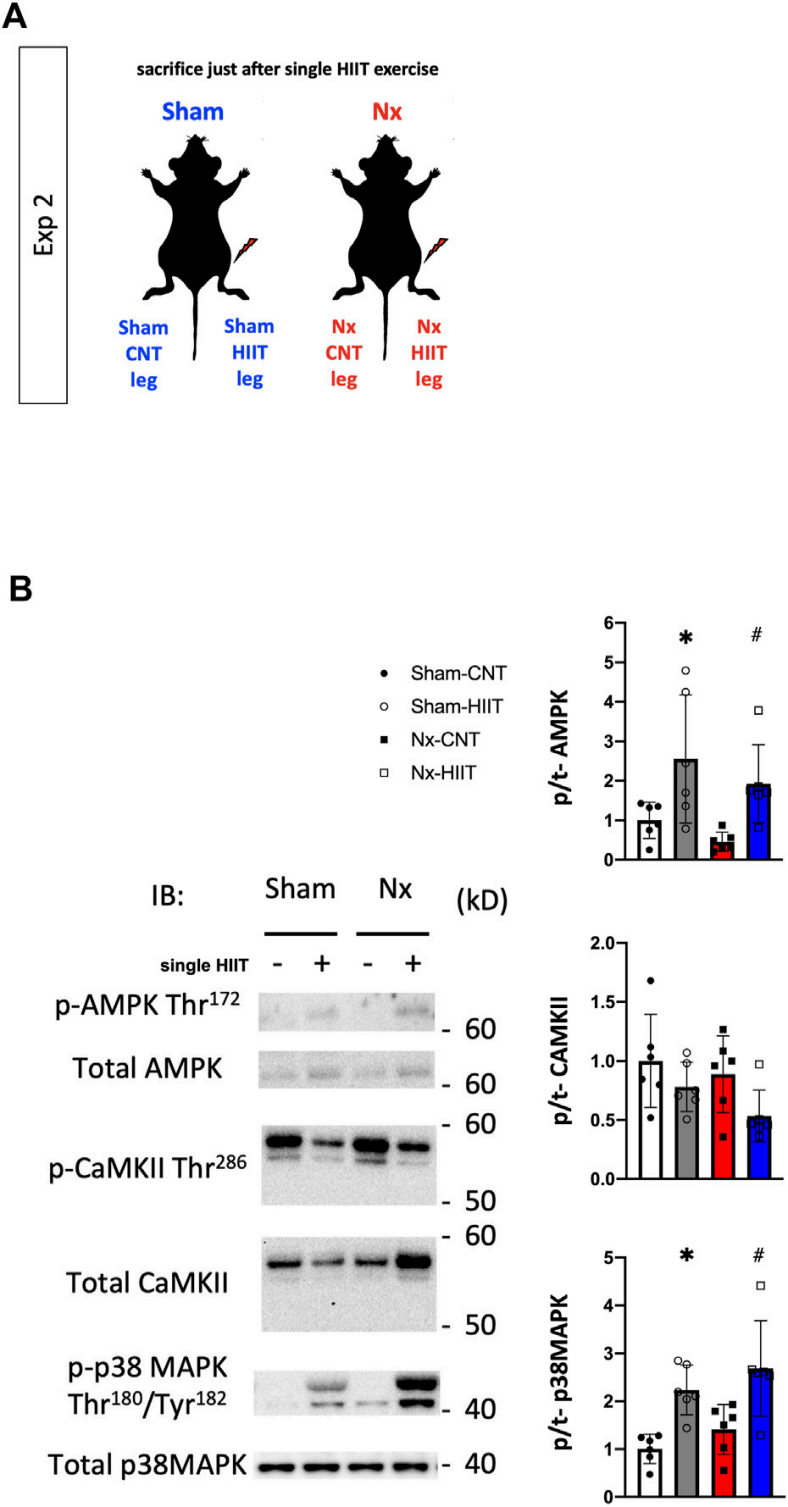
Phosphorylation levels of signaling proteins are increased after a single bout of high-intensity interval training (HIIT) with electrical stimulation. **(A)** In Exp 2, cellular signaling underlying the HIIT-induced physiological adaptations was investigated after an acute single bout of HIIT in both Sham and Nx rats. **(B)** Representative Western blots of the total levels and levels of phosphorylated AMPK Thr172, CaMKII Thr286, and p38 MAPK Thr180/Tyr182 in plantaris muscles from sham-operated (Sham) rats and 5/6 nephrectomy (Nx) rats with or without one bout of HIIT (*n* = 6 in each group). Two-way ANOVA followed by Tukey’s *post hoc* test was performed. *p < 0.05 vs. Sham-CNT, ^#^ *p* < 0.05 vs. Nx-CNT. Data are shown as means ± SD.

In experiment 2, to explore the signaling pathways activated by HIIT that enabled the mitochondrial adaptations observed so far, we tested phosphorylation reactions that occur with a single HIIT session in plantaris muscles from Sham and Nx rats. The phosphorylation levels of AMPK Thr172, CaMKII Thr286, and p38 MAPK Thr180/Tyr182 did not differ between the Sham-CNT and the Nx-CNT groups, while the phosphorylation levels of AMPK Thr172 and p38 MAPK Thr180/Tyr182 were increased immediately after one HIIT session compared to those in the CNT group (Figures 6A,B, *p* < 0.05).

## Discussion

While it has been clinically confirmed that CKD has been shown to be strongly associated with secondary sarcopenia, which results in severe muscle loss and weakness (Chatzipetrou et al., 2022), animal studies have further advanced our understanding by showing that muscle endurance is reduced at an early stage, even before muscle mass loss, and that this is accompanied by reduced mitochondrial function (Tamaki et al., 2014; Fusagawa et al., 2023). Recent CKD guidelines recommend exercise therapy for patients with CKD if the patient’s comorbidities and exercise tolerance allow systemic exercise therapy (Kidney Disease: Improving Global Outcomes KDIGO, 2022), but a safe and effective exercise protocol for muscle endurance has not been established. In the present study, we showed that HIIT with ES improves mitochondrial quantity and function in skeletal muscle and improves muscle endurance. To prevent the worst-case scenario of falls, fractures, and bedridden patients, our results offer the possibility of a promising therapeutic intervention that can be introduced to patients with severe renal failure who have limited physical activity.

Muscle endurance decline was observed and was ameliorated by the HIIT intervention in plantar flexor muscles of Nx rats within a few minutes from the onset of fatigue stimulation, suggesting the involvement of muscle endurance decline in fast-twitch muscles, as we previously reported. (Fusagawa et al., 2023). Intriguingly, it has also been shown that the force decline during phase 2 depends on mitochondrial dysfunction, (Lännergren and Westerblad, 1991; Stary and Hogan, 2000), and our results showed that the duration of torque maintained in phase 2 was clearly shorter in Nx rats and recovered with HIIT. Therefore, The results suggest that mitochondria have a vital role in muscle endurance in the areas reduced by CKD and improved by HIIT. The plantaris was selected for subsequent experiments because it is a more pure fast-twitch muscle fiber than the gastrocnemius, which contains a large amount of slow-twitch muscle in its deeper layers (Oishi et al., 2004). Although there is some literature that demonstrated the effect of ES on muscle hypertrophy, (Lotri-Koffi et al., 2019), HIIT with ES in this study did not ameliorate muscle mass in this CKD model. In our previous study, similar ES-HIIT (0.25 s stimulation/0.5 s) did not increase muscle mass in normal mice (Yamada et al., 2021). In contrast, ES training with a 2 s stimulation/6 s contraction cycle induced muscle hypertrophy in rat skeletal muscle (Ashida et al., 2018). Therefore, ES-HIIT may not have induced muscle hypertrophy because the mechanical stress was not sufficiently loaded due to the short duration of each contraction.

Muscle endurance also depends on fiber type, which is mostly defined by the MHC isoform, although the metabolic phenotype sometimes may differ (Blemker et al., 2024). Electrophoresis of MHC isoforms showed that MHC2b fiber with less mitochondrial content was increased in the Nx plantaris muscle and improved by HIIT, being consistent with changes in CS activity and the mitochondrial markers COX IV and prohibitin. The MHC type transformation described in CKD varies between reports and has not been conclusively established. Rats after 5/6 nephrectomy showed slow-to-fast fiber-type transformation with decreased oxidative capacity and increased glycolytic capacity in the tibialis anterior, a fast-twitch muscle (Acevedo et al., 2015). On the other hand, a slow to fast fiber-type transformation has been reported in muscle obtained from hemodialysis patients (Lewis et al., 2012). Tamaki et al., (2014) show opposing myofiber-type changes in a mouse model of CKD, and they argue that this difference may be due to the elimination of urea toxins by dialysis, whereas renal failure itself results in a slow to fast fiber-type transformation. Therefore, HIIT with ES in this study may also have an impact on the pathogenesis of urinary toxin accumulation due to CKD.

In the next step, we analyzed mitochondria, which have a significant impact on muscle endurance. The results of analysis of CS activity and the protein levels of VDAC1 and COXVI indicate that the mitochondrial mass, which was reduced in CKD, was ameliorated by HIIT with ES. In addition, the Flux analyzer showed that HIIT with ES improved respiratory capacity per mitochondrial protein as well as increasing protein levels of the respiratory chain complexes themselves, including NDUFS1, SDHB, and MTCO1, in mitochondria. Such effects of exercise have been reported in humans (Greggio et al., 2017), and similar results have been obtained in various conditions of mice with HIIT intervention using ES (Yamada et al., 2021; Yamada et al., 2022; Yamauchi et al., 2023). The results of this study showed that HIIT with ES had an enhancing effect on the mitochondrial supercomplex constitutions, which are reduced in CKD rats.

We confirmed a single effect of HIIT with ES and observed that phosphorylation of AMPK and p38 MAPK was enhanced. These two are known to be upstream of peroxisome proliferator-activated receptor γ co-activator-1 α and promote mitochondrial biogenesis by stimulating its activation (Egan and Zierath, 2013). AMPK is also known to be an upstream factor that promotes the organization of the mitochondrial supercomplex (Han et al., 2022). Interestingly, Nakamura et al. recently reported that treatment with sodium–glucose cotransporter-2 (SGLT2) inhibitors, which have been shown to significantly attenuate the progression of CKD (Herrington et al., 2023), improved muscle endurance via AMPK activation in a diabetic murine model (Nakamura et al., 2023). This finding suggests that activation of AMPK by HIIT with ES is at least one of the upstream signals that improve muscle endurance, promoting mitochondrial biogenesis and supercomplex formation in muscle with CKD-induced cachexia.

Interventions using electrical stimulation have long been used in patients with renal failure and chronic diseases (Schardong et al., 2020). Although there have been reports of some degree of muscle hypertrophy and improvement in single muscle tension, the protocols for electrical stimulation have not been optimized and muscle endurance has not been adequately evaluated. Some reports have suggested the effectiveness of systemic HIIT for CKD patients but have questioned whether patients could actually perform the protocol at sufficient intensity (Billany et al., 2022). Although exercise therapy has been recommended recently even for dialysis patients, it is still challenging to provide exercise therapy for patients who are unable to perform conventional dynamic training due to position limitations, hemodynamic instability during dialysis, low motivation, or fatigue (Cha and Lee, 2021). In this context, we proposed a new electrical stimulation protocol that shows improvement in muscle endurance. Dialysis patients need bed rest for several hours during dialysis, and providing safe and effective exercise therapy during that time is expected to compensate for the negative aspects of conventional dialysis therapy that may promote disuse muscle atrophy. Importantly, in voluntary exercise, fast-twitch muscle fibers are less likely to be recruited than slow muscle fibers because of the size principle (Henneman, 1957), but electrical stimulation would favor the activation of fast-twitch fibers in addition to the slow-twitch fibers even at relatively low levels of evoked force (Gregory and Bickel, 2005), making it possible to efficiently approach fast-twitch muscle, which is more vulnerable to cachexia. In addition, voluntary exercise involves alternation in the recruitment of motor units during the bout, whereas electrical stimulation causes the same motor units to continue firing, which can easily fatigue the muscle and induce metabolic adaptations (Bickel et al., 2011). ES therapy has also been reported to activate GABA and descending pain suppression mechanisms and can be used at high intensities after gradual increases in intensity (Chimenti et al., 2018). Therefore, we believe that ES therapy is effective for fast-twitch muscles, which are also vulnerable to CKD-related cachexia (Fusagawa et al., 2023; Wang et al., 2023), and that loading HIIT with ES at gradually increasing intensity is a feasible therapeutic intervention for CKD patients. The renal function assessment indicates that the 5/6 nephrectomy model in this study is in a state of severe renal failure (Besseling et al., 2021). The present study shows that it can be performed without worsening renal function in general condition as well as the previously used ES protocols (Dalise et al., 2017; Lotri-Koffi et al., 2019) and improves muscular endurance.

This study has the limitation that it does not compare with existing intervention methods according to the severity of renal insufficiency. However, exercise therapy is recommended regardless of the severity of renal failure as reported by Valenzuela et al., (2024) and it is significant to present an intervention that can be used even in patients with severely impaired exercise function. On the topic of cachexia, the 5/6 nephrectomy model of CKD has recently been called into question (Lair et al., 2024). Even though the model in this study shows muscle atrophy and weight loss, it will be essential to evaluate the presence of inflammation and increased basal energy expenditure in the future with respect to the 5/6 nephrectomy model, which is widely used as a model for this type of cachexia (Bowen et al., 2015). In addition, it has not been determined in this study whether changes in supercomplex formation are important or simply changes in the overall mitochondrial respiratory complex proteins. We would like to further study the effects of supercomplex formation and increase the significance of the effects of HIIT in the future.

## Conclusion

We have demonstrated that our HIIT protocol with ES can improve impaired muscle endurance in the skeletal muscle of a rat model of CKD with restoration of decreased mitochondrial content and mitochondrial dysfunction by activating AMPK and p38 MAPK signaling. Our findings highlight a protocol using electrical stimulation for safe and effective exercise therapy to restore muscle endurance in CKD patients.

## Data Availability

The original contributions presented in the study are included in the article/Supplementary Material, further inquiries can be directed to the corresponding author.
